# Multiple Cranial Organ Defects after Conditionally Knocking Out *Fgf10* in the Neural Crest

**DOI:** 10.3389/fphys.2016.00488

**Published:** 2016-10-25

**Authors:** Tathyane H. N. Teshima, Silvia V. Lourenco, Abigail S. Tucker

**Affiliations:** ^1^Department of Stomatology, School of Dentistry, University of Sao Paulo São Paulo, Brazil; ^2^Department of Craniofacial Development and Stem Cell Biology, King's College London London, UK

**Keywords:** *Fgf10*, ocular glands, thyroid, palate, cranial glands, CVP

## Abstract

*Fgf10* is necessary for the development of a number of organs that fail to develop or are reduced in size in the null mutant. Here we have knocked out *Fgf10* specifically in the neural crest driven by *Wnt1cre*. The *Wnt1creFgf10fl/fl* mouse phenocopies many of the null mutant defects, including cleft palate, loss of salivary glands, and ocular glands, highlighting the neural crest origin of the *Fgf10* expressing mesenchyme surrounding these organs. In contrast tissues such as the limbs and lungs, where *Fgf10* is expressed by the surrounding mesoderm, were unaffected, as was the pituitary gland where *Fgf10* is expressed by the neuroepithelium. The circumvallate papilla of the tongue formed but was hypoplastic in the conditional and *Fgf10* null embryos, suggesting that other sources of FGF can compensate in development of this structure. The tracheal cartilage rings showed normal patterning in the conditional knockout, indicating that the source of *Fgf10* for this tissue is mesodermal, which was confirmed using *Wnt1cre-dtTom* to lineage trace the boundary of the neural crest in this region. The thyroid, thymus, and parathyroid glands surrounding the trachea were present but hypoplastic in the conditional mutant, indicating that a neighboring source of mesodermal *Fgf10* might be able to partially compensate for loss of neural crest derived *Fgf10*.

## Introduction

*Fgf10* is an essential signaling molecule from the fibroblast growth factor family and is involved in the development of many organs, signaling through *Fgfr2b* in the epithelium (Ohuchi et al., 2000). Patients with mutations in one copy of the *Fgf10* ligand or its receptor have Lacrimo Acoustic Dental Digital (LADD) syndrome (OMIM 149730) or the related Aplasia of Salivary and Lacrimal Gland (ASLG) syndrome (OMIM 180920), characterized by defects in a variety of cranial glands (Rohmann et al., 2006). Mice with a complete knockout of *Fgf10* die at birth due to a complete lack of lungs and limbs and formation of a cleft palate (Min et al., 1998; Sekine et al., 1999; Rice et al., 2004). As in patients, loss of *Fgf10* also impacts on the development of a number of cranial glands, with null mutants showing complete loss of the salivary glands, thyroid gland, pituitary gland (Ohuchi et al., 2000), ocular glands (Govindarajan et al., 2000; Makarenkova et al., 2000) and the circumvallate papilla (CVP) housing the Von Ebner's glands in the tongue (Petersen et al., 2011). The salivary glands have been shown to arrest at the prebud stage, with heterozygous mice showing a delay in development that leads to later gland hypofunction (Jaskoll et al., 2005; May et al., 2015). The pituitary gland starts to initiate in the *Fgf10* null with an infolding of the oral epithelium to form Rathke's pouch at the back of the mouth, but the ectoderm is associated with high levels of apoptosis and the pouch disappears by E13.5 (Ohuchi et al., 2000). Other glands form but are reduced in size, such as the thymus glands (Ohuchi et al., 2000; Revest et al., 2001), and more subtle defects are also observed in the inner ear, in the patterning of the trachea cartilage rings, in the teeth and in the skin and hair follicles (Ohuchi et al., 2000; Pauley et al., 2003; Sala et al., 2011). In these organs loss of *Fgf10* may be compensated for by the presence of *Fgf7* or *Fgf3*, which can both bind to the same receptor (Zhang et al., 2006). In keeping with this loss of both *Fgf10* and *Fgf3* leads to a more severe defect in the inner ear (Wright and Mansour, 2003), and knockout of the receptor *Fgfr2b* leads to additional defects not observed in *Fgf10* knockouts, such as arrest of tooth development at the bud stage (De Moerlooze et al., 2000).

During development *Fgf10* is expressed in the mesenchyme that surrounds many developing organs (lungs, limbs, ocular glands, palatal shelves, salivary glands (Bellusci et al., 1997; Moustakas et al., 2011; Wells et al., 2013). In contrast its receptor, Fgfr2b, is expressed in epithelial structures overlying these regions, emphasizing the importance of epithelial-mesenchymal interactions (Peters et al., 1992; Orr-Urtreger et al., 1993; Rice et al., 2004). In the developing brain *Fgf10* is expressed in the infundibulum, which signals to the developing oral epithelium during pituitary gland development (Takuma et al., 1998). Early on during facial development *Fgf10* is expressed in the oral epithelium of the first pharyngeal arch (Kettunen et al., 2000; Wells et al., 2013), with expression also observed in the tooth germ epithelium in some species (Moustakas et al., 2011). In the otic region *Fgf10* is first expressed in the mesoderm derived mesenchyme around the otic epithelium at E8.75 and then in the otic cup and otic vesicle at E9 and E9.5 (Wright and Mansour, 2003). *Fgf10* is therefore expressed in a range of tissues during development.

In this paper we have conditionally knocked out *Fgf10* specifically in neural crest derived tissue using the *Wnt1*cre transgenic line (Chai et al., 2000). Previously a conditional knockout of *Fgf10* has been carried out using *Dermo1* cre, which led to specific loss of *Fgf10* in the mesoderm around the developing lungs, resulting in lung branching defects (Abler et al., 2009). By knocking out *Fgf10* in neural crest derived tissues only we aim to investigate which phenotypes in the null mutant are a consequence specifically of *Fgf10* expression in the neural crest. A number of tissues in the head are known to be derived from the neural crest. These include the mesenchyme around the developing salivary glands, thyroid and thymus glands, teeth, and the palatine bone (Chai et al., 2000; Jiang et al., 2000; Jaskoll et al., 2002; Müller et al., 2008; Johansson et al., 2015). The *Fgf10* expressing mesenchyme that underlies the forming CVP in the tongue is also neural crest derived (Hosokawa et al., 2010). The origin of the tissue around the developing ocular glands has not been confirmed as the developing eye is surrounded by neural crest derived mesenchyme and lateral plate mesoderm, which together forms the periocular mesenchyme (Langenberg et al., 2008). In contrast the limbs and lungs are surrounded by mesoderm and so would be predicted to develop normally in the conditional *Wnt1cre Fgf10* mice. The pituitary would also be expected to be normal in these conditional mutants as the source of the *Fgf10* is the neuroectodermal infundibulum (Takuma et al., 1998). In addition we compare the conditional knockout to the phenotype in the null *Fgf10* mouse to clarify the role of neural crest derived *Fgf10* in a variety of craniofacial tissues, and identify a few discrepancies with the published literature.

## Materials and methods

### Transgenic mice

*Fgf10 floxed* (Fgf10A02 tmc1c) mice on a C57Bl6 background were produced by MRC-Harwell as part of the International Mouse Phenotyping Consortium (IMPC; Pettitt et al., 2009; Skarnes et al., 2011; Bradley et al., 2012). *Fgf10fl/fl* females were crossed to *Wnt1cre/Fgf10 fl/*+ males to generate *Wnt1creFgf10fl/fl* embryos (3 litters), collected at E14.5, E15.5, and E19.5 (E14.5 *n* = 3; E15.5 *n* = 3; E19.5 *n* = 2). These conditional mutants were compared to *Fgf10fl/fl* littermates that did not carry the cre and were therefore phenotypically wildtype. A total of 6 *Fgf10* null embryos generated on a mixed C57Bl6/CD1 background (E14.5, E15.5, E18.5) were used to compare the conditional phenotype with that of the complete null.

*Wnt1cre* males were mated to *tdTomato* reporter females (Gt(ROSA)26 Sor tm14(CAG-tdTomato)Hze JAX labs) to lineage trace the neural crest and were viewed with a Nikon SMZ25 fluorescence microscope.

The *Wnt1cre* mouse is widely used for neural crest specific knockout studies, however, it has been linked to elevated levels of Wnt signaling in the midbrain, particularly in *Wnt1cre* Tg/Tg embryos (Lewis et al., 2013). We used *Wnt1cre* Tg/+ mice for our crosses to reduce this effect. In addition, no facial phenotype was observed in *Wnt1cre* embryos compared to WT littermate controls (data not shown), agreeing with results that show that any midbrain dysmorphologies caused by the *Wnt1cre* line do not cause cranial shape changes (Heuze et al., 2014).

Pregnant mice were culled using schedule 1 culling methods at E14.5 to E19.5, just prior to giving birth. All procedures were carried out as agreed by the UK Home Office and King's College London. Animals were housed in approved non-specific-pathogen-free conditions. Animal experiments conform to ARRIVE (animal research: reporting of *in vivo* experiments) guidelines.

Embryos were photographed using a Leica dissecting microscope.

### Skeletal preps

E19.5 embryos were skinned and eviscerated before fixing in 95% Ethanol. Samples were then stained in alcian blue and alizarin red to stain cartilages and bones, respectively. Embryos were cleared in 0.5% KOH and stored in glycerol and photographed using a Leica dissecting microscope.

### Histology

Embryos were fixed in 4% paraformaldehyde and dehydrated through an ethanol or methanol series before embedding in wax. Sections were cut on a microtome at 8 μm and slides were stained with a trichrome stain (Haematoxylin, alcian blue and sirrus red). Sections were photographed using a Nikon microscope.

### Thymus analysis

To compare the size of the thymus glands in the conditional mutants the thymus glands from 3 *Fgf10fl/fl* mice and 4 *Wnt1cre Fgf10fl/fl* mice were assessed using histology sections at E14.5. The number of sections with a thymus was multiplied by the thickness of the sections (8 μm) to give the total extent of the gland. This was then compared using a student *t*-test where significance was *P* < 0.05.

### Radioactive *in situ* hybridisation

CD1 mice were used for expression of *Fgf10*. *Fgf10* probe was a gift from Ivor Mason. *In situ* hybridisation on wax sections was carried out according to previously published protocols (Kettunen et al., 2000). *Fgf10* antisense probe was synthesized using 35S labeled UTP and signal was identified using sliver emulsion, which when developed showed positive signal as white grains under darkfield. The magic wand tool in photoshop was used to pseudocolour the white grains red and this layer was overlain on top of the light field image to produce a final compound image.

## Results

### Normal lung, limb and pituitary development but defective palate formation in *Wnt1cre Fgf10* conditional mutants

**Figure 1:**
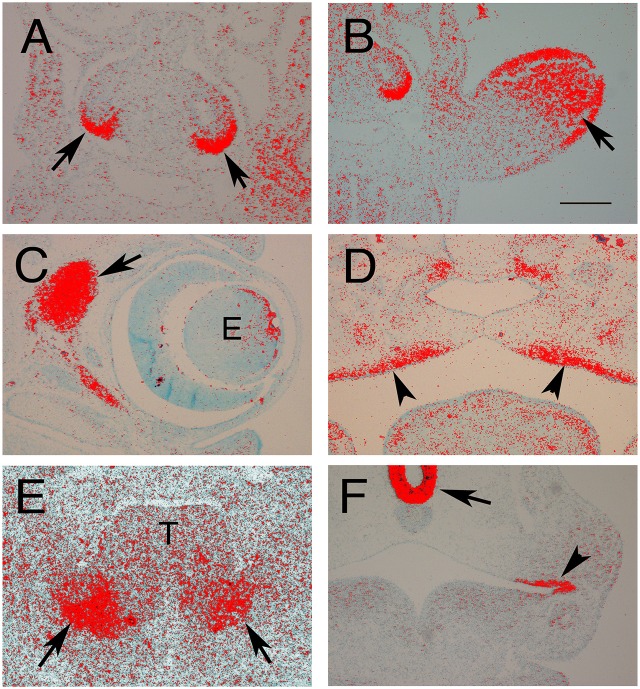
**Expression of ***Fgf10*** in the developing embryo. (A,B,F)** E10.5 Frontal sections. **(C,D)** E15.5 frontal sections. **(E)** E12.5 Frontal section. Red grains indicate signal. **(A)** Developing lungs. *Fgf10* is expressed around the tips of the epithelial lung buds (arrows). **(B)** Developing Limb. *Fgf10* is expressed in the mesenchyme in the distal part of the limb (arrows). **(C)** Developing eye (e). *Fgf10* is expressed in the mesenchyme at the back of the eye (arrow). **(D)** Developing palate. *Fgf10* is expressed in the mesenchyme adjacent to the palatal epithelium (arrowsheads). **(E)** Developing salivary gland. *Fgf10* is expressed in the mesenchyme (arrows) underlying the epithelial buds on either side of the tongue (T). **(F)** Developing pituitary and molars. *Fgf10* is expressed at the base of the brain (arrow) underlying rathke's pouch and in the oral epithelium (arrowhead). Scale bars in **(B)** = 200 μm, same scale **(A,C–F)**.

**Figure 2:**
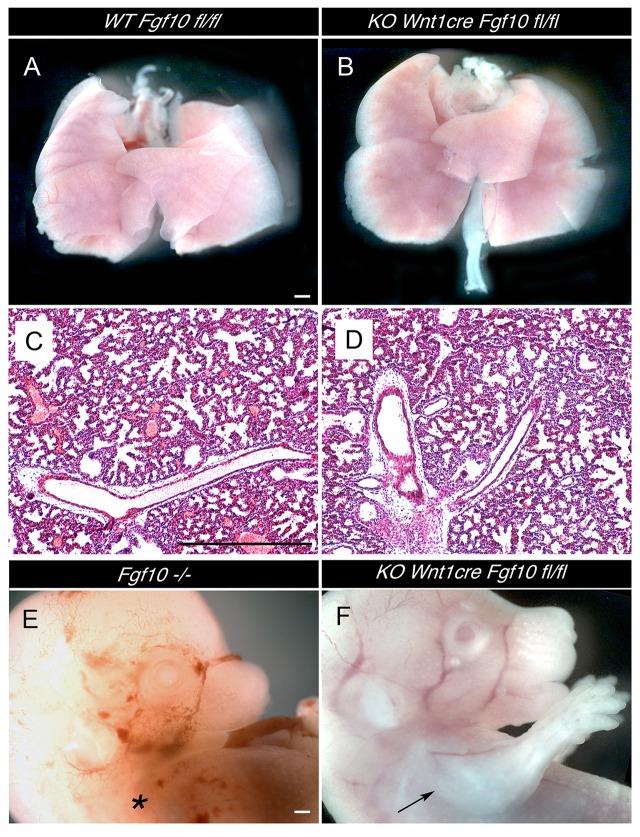
**Normal development of lungs and limbs in ***Wnt1creFgf10*** mutant mice. (A,C)** *Fgf10 fl/fl* Control. **(B,D,F)** *Wnt1creFgf10 fl/fl* mutant. **(E)** Fgf10 null mutant. **(A,B)** Dissected lungs E19.5. **(C,D)** Lung histology at E19.5. **(E)** Absence of limbs in null *Fgf10* mutant at E14.5 (^*^). **(F)** Normal limb development in the conditional mutant (arrow). Scale bars: 500 μm.

In agreement with previous studies we observed expression of *Fgf10* in the developing lung, limb, ocular glands, palate, salivary glands, and epithelium of the developing maxilla and mandible (Figure 1). As expected conditional mutants (*Wnt1creFgf10fl/fl*) had normally developing lungs at E19.5 (Figures 2A,B; *N* = 2/2). Histology of the lungs matched that of littermate controls (*Fgf10fl/fl*; Figures 2C,D), as although there are *Wnt1cre* positive cells located within the developing lungs these are associated with the intrinsic nervous system, which does not express *Fgf10* (Freem et al., 2010). The limbs also formed normally (*N* = 8/8), in contrast to complete loss of these structure in *Fgf10* null mutants (Figures 2E,F; *N* = 6/6).

**Figure 3:**
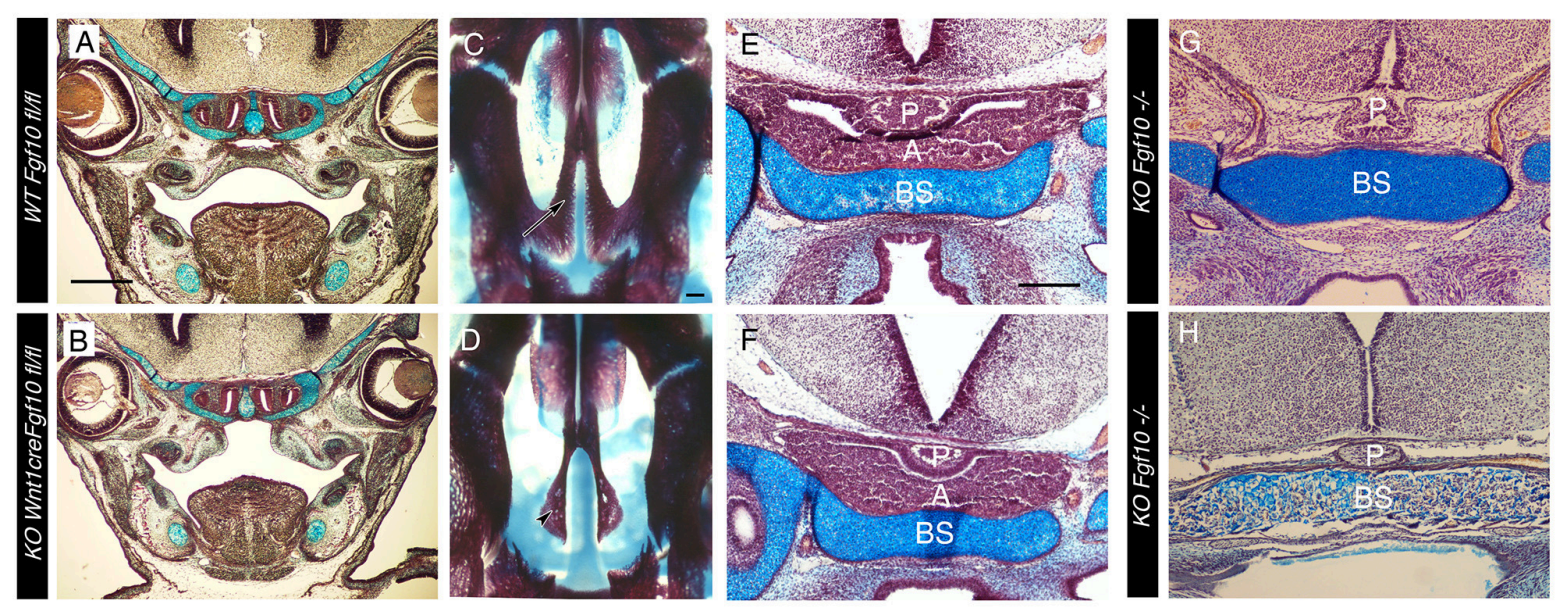
**Cleft palate but normal pituitary formation in ***Wnt1creFgf10*** mutant mice. (A,C,E)** *Fgf10 fl/fl* Control. **(B,D,F)** *Wnt1creFgf10* mutant. **(G,H)** *Fgf10* −/−. **(A,B)** Frontal histology sections through the palate at the level of the molars at E15.5. The palatal shelves have not formed correctly in the conditional mutant. **(C,D)** Skeletal preps of the palate at E19.5. Arrow in C points to the WT palatal processes of the maxilla that have met in the midline. These processes are missing in the conditional mutant and the underlying vomer (arrowhead) is visible. **(E–H)** Developing pituitary gland. **(E,F)** The anterior and posterior lobes form as normal in the conditional mutant at E15.5. **(G,H)** The anterior lobe is missing but the posterior lobe is still evident in the *Fgf10* null at E15.5 **(G)** and E18.5 **(H)**. A = anterior lobe derived from oral ectoderm. P = Posterior lobe derived from neuroepithelium. BS = Basisphenoid. Scale bars in **(A,B)** = 500 μm. Scale bars **(C,D)** = 200 μm. Scale bars **(E–H)** = 200 μm.

In the palate *Fgf10* is expressed in the mesenchyme adjacent to the oral epithelium (Figure 1D). Palate development was disrupted in the conditional mutants with a failure in development of the palatal shelves at E15.5 (Figures 3A,B; *N* = 3/3), suggesting problems in shelf development similar to those observed in the null (Rice et al., 2004). Just before birth (E19.5) defects in formation of the palatine processes of the maxilla and palatine bone were clear, leaving the vomer visible when viewed from the oral side (Figures 3C,D; *N* = 2/2). Due to the cleft palate the conditional mutant would not be predicted to survive past birth, and, in agreement with this, no mutants were discovered at P1 (postnatal day 1) in one litter where the mother was left to litter down.

In the *Fgf10* null the ectoderm part of the pituitary (Rathke's pouch), which forms the anterior lobe, is completely lost due to high apoptosis at early stages of development (Ohuchi et al., 2000). In contrast the posterior lobe, which is derived from the neuroectoderm, is apparent at E13.5 but regresses in the absence of the anterior lobe and has been reported to be lost by E15.5 (Ohuchi et al., 2000). In agreement with the published data, we observed a complete loss of the ectodermally derived portion of the pituitary at E15.5 in the *Fgf10* null mice, but the posterior lobe was still present at this stage (Figure 3G). It was also still evident at E18.5 (Figure 3H), suggesting that this part of the pituitary is not dependent on the presence of the anterior lobe as previously proposed. The conditional mutant showed normal development of both the anterior and posterior lobe of the pituitary at E15.5 (Figures 3E,F), indicating no requirement for neural crest derived Fgf10 in its formation.

### Development of a hypoplastic CVP and loss of salivary and ocular glands in *Wnt1cre Fgf10* conditionals

**Figure 4:**
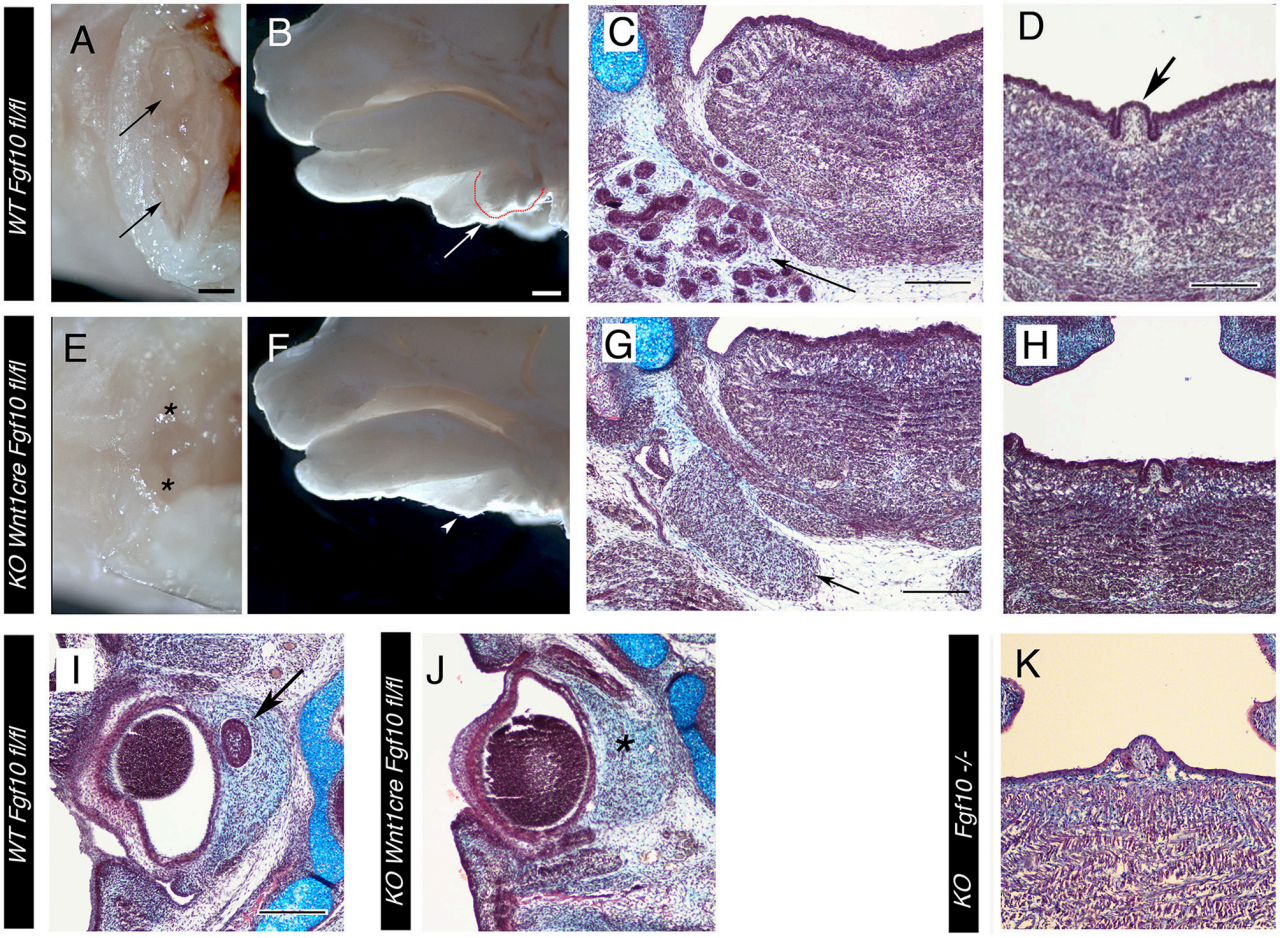
**Loss of Salivary and ocular glands in ***Wnt1creFgf10*** mutant mice. (A–D,I)** *Fgf10 fl/fl* Control. **(E–H,J)** *Wnt1creFgf10 fl/fl* mutant. **(K)** *Fgf10* −/−. **(A,E)** E15.5 head ventral view looking up at the paired submandibular and sublingual glands (arrow). The glands are missing in the mutant (asterix). **(B,F)** E15.5 head dissected down the middle to reveal the glands at the base of the tongue, outlined in red and arrowed. The glands are missing in the conditional mutant (arrowhead). **(C,D,G,H,K)** Frontal sections at the level of the tongue at E15.5. **(C,G)** The branching epithelial tissue of the salivary gland is observed in the control (arrow) but are missing in the mutant. The mesenchymal capsule, however, is still evident (arrow). **(D,H,K)** Posterior tongue showing the single circumvallate papilla (CVP) in the center (arrow in **D**). **(H,K)** A CVP formed in the null and conditional but was smaller than in the WT. **(I,J)** Frontal sections of the eye at E15.5. The harderian gland at the back of the eye failed to form in the conditional mutant (asterix in **J**). Scale bars **(A,B)** = 500 μm, same scale in **(E,F)**. Scale bars **(C,D,G,I)** = 200 μm, same scale in **(H,J,K)**.

Salivary glands are absent in *Fgf10* nulls. In keeping with this result the salivary glands were completely absent in the conditional mutant (Figures 4A–C,E–G; *N* = 8/8), although a mesenchymal capsule still formed despite the lack of any branching epithelium (Figure 4G), phenocopying the null phenotype (Wells et al., 2013). These results are in agreement with the neural crest origin of the salivary gland mesenchyme (Jaskoll et al., 2002).

Slightly unexpectedly, the conditional mutants also formed a circumvallate papilla (CVP) at the back of the tongue (Figures 4D,H; *N* = 3/3). The CVP was smaller in size compared to littermate controls and the two fingers of invaginating epithelium were reduced, similar to the phenotype observed in *Eda* pathway mutants (Wells et al., 2011). We checked the development of the CVP in *Fgf10* null embryos, where the CVP has been recorded as missing, and found that the CVP was present but reduced in size in the *Fgf10* null embryos at E15.5 (*N* = 3/3), similar to the phenotype in the conditional knockout, indicating that the CVP can initiate in the absence of *Fgf10* (Figure 4K).

*Fgf10* is expressed at high levels in the mesenchyme around the developing eye during the stages of ocular gland development (Figure 1C). At E15.5 the Harderian gland had initiated in littermate controls at the back of the eye, while this gland was missing in the conditional mutant, despite the presence of a mesenchymal capsule (Figures 4I,J; *N* = 3/3). This is in agreement with previous research that *Fgf10* expression is essential for the formation of ocular glands, and confirms that the source of *Fgf10* is the neural crest around the eye.

### Hypoplasia of neck glands but normal tracheal cartilage patterning in conditional mutants

**Figure 5:**
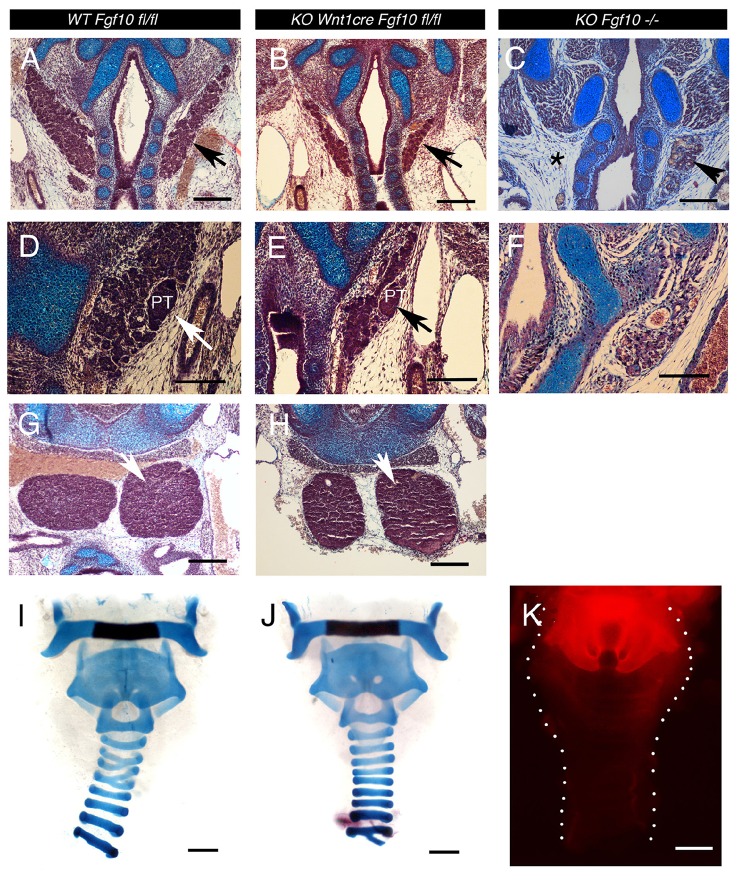
**Cranial gland and trachea development in the ***Wnt1creFgf10*** mutant mice. (A,D,G,I)** *Fgf10 fl/fl* Control. **(B,E,H,J)** *Wnt1creFgf10* mutant. **(C,F)** *Fgf10* −*/*− null mice. Frontal sections E15.5. **(A,B,C)** Developing thyroid glands (arrowed) A severely hypoplastic gland was observed in the null mutant but was missing on one side (asterix). **(D,E,F)** Developing parathyroid glands (arrows). No parathyroid was observed in the Fgf10 null. **(G,H)** Developing Thymus glands (arrow). **(I,J)** Alcian blue staining for tracheal cartilages at E19.5. Dissected trachea show normal morphology with formation of cartilage rings. **(K)** The neural crest, as marked by red in *Wnt1cre/tdTom* mice, does not extend down the trachea past the thyroid cartilage as shown at Postnatal day 0. PT = parathyroid. Scale bars **(A–C,G,H)** = 200 μm. Scale bars **(D–F)** = 100 μm. Scale bars **(I–K)**: 500 μm.

The thyroid gland was present, but reduced in size in the conditional mutants (Figures 5A,B; *N* = 3/3). This suggests either that not all *Fgf10* signaling required for formation of this gland is neural crest derived, or that in fact this gland can develop in the absence of *Fgf10.* To confirm this we looked at development of the thyroid in *Fgf10* null mutants. A small thyroid was observed in 2/3 cases, and in both cases was unilateral, indicating that the thyroid is able to initiate in the absence of *Fgf10* (Figure 5C). The gland tissue was located in the correct place, under the cricoid cartilage, indicating that migration cues were unaffected in the mutant, however the gland did not extend as far anteriorly toward the thyroid cartilage. The null gland when present was smaller than that observed in the conditional mutant suggesting another source of Fgf10 might be available for development of this gland in the conditional mutant. Alternatively development of this gland might depend on interaction with other tissues, not affected in the conditional. As the parathyroids migrate to the thyroid we checked for the presence of these glands in our samples. The parathyroids were normally positioned next to the thyroid in the conditional mutant, but as with the thyroid were slightly hypoplastic (Figures 5D,E). No evidence of parathyroids were observed in the *Fgf10* null mutant mice (*N* = 3; Figures 5F), suggesting again an alternative non-neural crest source for parathyroid gland development in our conditional mutants. The thymus glands in the *Fgf10* null mutants have been shown to be hypoplastic (Ohuchi et al., 2000; Revest et al., 2001). In the conditional mutants the thymus glands were present (Figures 5G,H) but appeared slightly reduced in size in the conditional mutant at E15.5, although analysis at E14.5 showed no statistically significant difference (*P* = 0.684). This is in contrast to the null where the thymus glands are much smaller by this stage (Revest et al., 2001). In each case, therefore, the neck glands were less severely affected in the conditional compared to the null mutant.

We therefore decided to confirm the position of the boundary between the neural crest and mesoderm in this region of the neck. Tracheas from *Wnt1cre-tdTom* reporter mice were dissected out with the glands removed at P0 to identify the limit of the neural crest, which was found to lie between the thyroid (*Wnt1* positive) and cricoid (*Wnt1* negative) cartilages, with the tracheal rings being mesodermal (Figure 5K). This therefore places the thymus, thyroid and parathyroids within the mesoderm, despite the glands themselves having a neural crest origin. In the *Fgf10* null the trachea cartilages are severely mispatterned (Sala et al., 2011). We therefore investigated tracheal cartilage formation at E19.5 by skeletal prep in the conditional mutants. As expected, given the limit of the neural crest in this region, the cartilage rings were unaffected in the conditional mutants (*N* = 2/2), matching the pattern in littermate controls (Figures 5I,J).

## Discussion

The development of the ocular and submandibular and sublingual salivary glands was completely dependent on *Fgf10* signaling from the neural crest derived mesenchyme, with development arresting at early initiation stages as in the null. This paper therefore confirms that the *Fgf10* expressing ocular and salivary gland mesenchyme is derived from the neural crest. As expected, palate development was also disrupted after loss of *Fgf10* in the neural crest derived mesenchyme of the developing palatal shelves, and the conditional mutation is likely to cause lethality.

In contrast to these cranial glands other more posterior glands, such as the thyroid, thymus, and parathyroids did not phenocopy the complete loss of *Fgf10*. The thymus, parathyroids and thyroid initiate within neural crest derived mesenchyme (Müller et al., 2008; Johansson et al., 2015) and then migrate more posteriorly to sit within mesodermally derived mesenchyme, as supported by our neural crest lineage analysis of the trachea, and previous lineage tracing that mentions the tracheal rings are not neural crest derived (Matsuoka et al., 2005). All of these glands are severely affected in the *Fgf10* null but the conditional mutants had a milder phenotype. In all three glands it is possible that other Fgfs and alternative signaling pathways are able to compensate for the initial loss of *Fgf10* in this tissue, allowing their initiation, while mesodermal *Fgf10* may be able to act once the glands have reached their final positions in the neck. In agreement with this *Fgf10* is expressed in the mesenchyme around the thymus at E13.5, a stage after the glands have reached their final position (Revest et al., 2001), and is strongly expressed in the ventral mesenchyme of the developing trachea from E14.5 (Sala et al., 2011). Mesodermal Fgf10 is therefore in the right place to be able to signal to the more posterior glands. It is also possible that signals between these tissues and other neighboring structures are important for their development, and that their presence is interdependent.

Early on in development *Fgf10* is strongly expressed in the oral epithelium. It was therefore possible that some of the orally derived structures would have a reduced phenotype when compared to the *Fgf10* null. The glands of the oral cavity, however, appeared to mimic the null phenotype indicating that only the loss of *Fgf10* in the neural crest was critical. Moreover the epithelial expression appeared to have no influence on the developing teeth, the molars having only a very minor defect in relative size similar to the null (Ohuchi et al., 2000). It would therefore be interesting to see whether knocking out *Fgf10* in the early epithelium has any effect on development of these key structures. As expected development of the pituitary, limbs and lungs was normal in the conditional knockout, in which *Fgf10* was provided by the neuroepithelium and mesoderm rather than neural crest. The tracheal rings were also normal highlighting the fact that the *Fgf10* expressing mesenchyme that forms the cartilage rings is not neural crest derived.

Our comparison of the conditional mutants with the *Fgf10* null mutants revealed a few differences between the phenotype observed in our null mice and in previously published data. For example, although it has been reported that the thyroid fails to form in *Fgf10* null mice (Ohuchi et al., 2000) in our null mice a small amount of glandular tissue was present around the trachea in the region of the thyroid but this was only observed unilaterally. Interestingly, in the Ohuchi paper although the text states no gland forms the figures highlight a rudimentary thyroid. The thyroid therefore does appear to be able to form in the complete absence of *Fgf10* but is severely hypoplastic, while we saw no evidence of a parathyroid.

We also observed development of a hypoplastic CVP in the tongue, which had previously been reported as missing in the *Fgf10* mutant (Petersen et al., 2011). *Fgf7* is also expressed in the mesenchyme of the developing tongue and may compensate for the loss of *Fgf10* in this structure (Sohn et al., 2011). These differences with the published data may indicate variation due to genetic background. For our studies we investigated *Fgf10* nulls on a mixed C57Bl6/CD1 background, while other papers have used a mixed C57bl6/CBA or C57Bl6/129SVJ or not reported the background used (Min et al., 1998; Sekine et al., 1999; Ohuchi et al., 2000; Rice et al., 2004; Petersen et al., 2011). In fact the arrest in limb development was reported to occur at slightly different time-points when the *Fgf10* knockout was originally reported by two groups, with the difference being suggested to be due to genetic background (Min et al., 1998; Sekine et al., 1999). Our findings have therefore shed light on the structures affected by neural crest expressing *Fgf10* but have also revealed some differences in the published literature which merit further investigation.

## Ethics statement

All experiments were approved by the Home Office and conducted with the correct project and personal licenses. Experiments using GMOs were approved by the Kings Biological Safety Committee.

## Author contributions

AT and SL conceived the experiments, AT and TT conducted the experiments and undertook data acquision, AT, SL, and TT wrote the manuscript.

## Funding

TT was funded by São Paulo Research Foundation (FAPESP), grant 2015/02824-6. AT is funded by the Wellcome Trust (102889/Z/13/Z).

### Conflict of interest statement

The authors declare that the research was conducted in the absence of any commercial or financial relationships that could be construed as a potential conflict of interest. The reviewer CC and handling Editor declared their shared affiliation, and the handling Editor states that the process nevertheless met the standards of a fair and objective review.
